# Genome-wide evolutionary characterization and expression analysis of *SIAMESE-RELATED* family genes in maize

**DOI:** 10.1186/s12862-020-01619-2

**Published:** 2020-07-29

**Authors:** Zhengquan Zhang, Jianzhou Qu, Feifei Li, Silu Li, Shutu Xu, Renhe Zhang, Jiquan Xue, Dongwei Guo

**Affiliations:** 1 Key Laboratory of Biology and Genetic Improvement of Maize in Arid Area of Northwest Region, Ministry of Agriculture, College of Agronomy, Northwest A&F University, Yangling, 712100 Shaanxi China; 2 Maize engineering technology research center of shaanxi province, Yangling, 712100 Shaanxi China

**Keywords:** Endoreduplication, Genome-wide analysis, *ZmSMR* gene family, Gene expression, Maize, Endosperm, Stress tolerance

## Abstract

**Background:**

The *SIAMESE* (*SIM*) locus is a cell-cycle kinase inhibitor (CKI) gene that has to date been identified only in plants; it encodes a protein that promotes transformation from mitosis to endoreplication. Members of the SIAMESE-RELATED (SMR) family have similar functions, and some are related to cell-cycle responses and abiotic stresses. However, the functions of SMRs are poorly understood in maize (*Zea mays* L.).

**Results:**

In the present study, 12 putative SMRs were identified throughout the entire genome of maize, and these were clustered into six groups together with the SMRs from seven other plant species. Members of the ZmSMR family were divided into four groups according to their protein sequences. Various *cis*-acting elements in the upstream sequences of *ZmSMRs* responded to abiotic stresses. Expression analyses revealed that all *ZmSMRs* were upregulated at 5, 20, 25, and 35 days after pollination. In addition, we found that *ZmSMR9/11/12* may have regulated the initiation of endoreplication in endosperm central cells. Additionally, *ZmSMR2/10* may have been primarily responsible for the endoreplication regulation of outer endosperm or aleurone cells. The relatively high expression levels of almost all *ZmSMRs* in the ears and tassels also implied that these genes may function in seed development. The effects of treatments with ABA, heat, cold, salt, and drought on maize seedlings and expression of *ZmSMR* genes suggested that *ZmSMRs* were strongly associated with response to abiotic stresses.

**Conclusion:**

The present study is the first to conduct a genome-wide analysis of members of the ZmSMR family by investigating their locations in chromosomes, identifying regulatory elements in their promoter regions, and examining motifs in their protein sequences. Expression analysis of different endosperm developmental periods, tissues, abiotic stresses, and hormonal treatments suggests that *ZmSMR* genes may function in endoreplication and regulate the development of reproductive organs. These results may provide valuable information for future studies of the functions of the SMR family in maize.

## Background

Cell-cycle regulation is an important mechanism that allows multicellular organisms to adapt to various internal and external stimuli. This process is regulated by key cell-cycle regulatory proteins, including cyclin-dependent kinases (CDKs) and their corresponding cyclin (CYC) partners [1, 2]. The primary model of the cell cycle assumes that duplicated DNA undergoes equal division and is distributed into two daughter cells by mitosis and cytokinesis. A modified model assumes that endoreplication or endoreduplication involves multiple rounds of DNA replication without cell division, thereby resulting in polyploidization [3, 4]. However, our understanding of specific regulatory mechanisms for each component of the cell cycle remains limited.

Endoreplication occurs in various organs of approximately 90% of angiosperms [5]. Endoreplication occurs especially in tissues and cells with rapid proliferation and high rates of metabolic activity [6] such as antipodal cells, synergid cells of female gametophytes, suspensors, cotyledons, filaments, and tapeta [5], plumular axes of seedlings [7], stem and leaf epidermis [8], maize endosperm [4], sorghum endosperm [9], fruit mesocarp [10], and young leaves [11]. Endoreplication can promote petal development in the epidermis of *Arabidopsis thaliana* [12] and cabbage [8]; it also protects plants against various stressful environmental stimuli such as salt, heat, and cold [13–15]. *Arabidopsis* is more able to tolerate damage from water deficits when the endoreplication level increases in leaves [13]. Endoreplication is also a common pathway for cell growth during the development of cereal seeds such as rice, wheat, sorghum, and maize. At 8–10 days after pollination (DAP), the middle cells of the endosperm gradually turn to endoreplication. At 20 DAP, the DNA content of these cells reach 96–162 c, indicating that these cells may have undergone at least six rounds of DNA synthesis; in terms of the grain development process, the period of endoreduplication and the period of grain filling are almost completely synchronized, indicating a close relationship between kernel formation and endoreplication [16, 17]. In addition, at the late stage of endosperm development, endoreplication is also considered as a signal for the initiation of programmed cells death, which will contribute to the disintegration of endosperm cells and to release the large amount of nucleic acid and phosphoric acid that can be used for embryonic development and germination [18].

Endoreplication is a complex process, and its regulation is mainly achieved via the activity of cyclin-dependent kinase (CDK). When CDK activity is high, cells tend to undergo normal mitosis; with low activity, the cells transition to endoreplication. The activity of cdk is regulated by a variety of factors; it can be inhibited either by binding to chaperone protein-cyclin (CYC) to produce a heterodimer or by binding to a cyclin kinase inhibitor (CKI). *A-*, *B*-, and *D*-*CYC* are expressed in maize endosperm, and the peak CDK activities of CycB1;3, D5;1 and D2;1 occur at 11 DAP and are indicative of high mitotic and endoreplication activities [19, 20]. The major CDK inhibitors (CKI) include Wee1 [21] and three CKIs, namely KRP;1, KRP;2 [22], and RBR3 [23, 24]. The highest expression level of *Wee1* was observed during endoreplication, indicating that it may contribute to the inhibition of cell division [25]. KRP;1 and KRP;2 inhibit the related kinase activities of CycA1;3 and CycD5;1, which involves the regulation of the S phase [22]. In contrast, RBR1 inhibits RBR3 [23]. The expression of *RBR1* is constant and increases during the later stages of endoreplication. *RBR3* expression, however, is drastically reduced after commencement of endoreplication, indicating that it is involved in mitosis but that it is not essential to endoreplication. The upregulation of *RBR1* during endoreplication implies that it may regulate the conversion of the G and S phases during internal endoreplication [23].

*Arabidopsis* trichomes are considered as an ideal model for studying endoreplication [26]. Low levels of endoreplication can induce trichomes transformation into epidermal cells, even though these have entered the early stages of differentiation; in turn, high levels of endoreplication can induce epidermal cells to form trichomes [27]. The endoreplication level of *Arabidopsis* trichomes is regulated positively by the *SIM* gene, and *SIM* also is the only regulator found to date that exists only in plants [28]. In the *Arabidopsis SIM* mutation, the trichomes become a multicellular cluster with a single-cell branched shape, and the DNA content in each cell nucleus is significantly reduced [28]. The *SIM* gene encodes a cyclin kinase repressor (CKI) that initiates mitotic activity toward endoreplication by interacting with CYCD/CDK. The SIM is localized in the nucleus, and it interacts with one or more CYCD/CDDA complexes but not with cyclin CYCB or CDKB [29]. In maize, two SMR proteins have been found that contain the pest domain [30]; however, details concerning the expression and function of these proteins have not been documented.

In the present study, 12 members of the maize *SMR* gene family were identified by screening the GDB maize database. The protein character, promoter structure, chromosome localization, and protein motif and phylogeny were analyzed, and the results were employed to predict the roles of various cis-acting elements in the promoter regions of the ZmSMR genes in endogenous and stress responses. Finally, the expression differences of these members at the transcriptome level under different endosperm development stages, tissues, abiotic stresses, and hormonal treatments were determined. Collectively, our findings may contribute to our understanding of the role of SMR genes in the maize defense response and may also provide valuable information concerning their roles in endoreplication.

## Results

### Identification and nomenclature of SMR family members in maize

A total of 12 SMR family members were identified in the maize genome (Additional file 1), hereby designated as ZmSMR1–12 (Table 1). Among the identified ZmSMRs, ZmSMR11 was the largest protein, with 152 amino acids (aa), whereas ZmSMR1 was the smallest, with 90 aa. The molecular weights of the 12 members ranged from 9272.63 Da (ZmSMR2) to 16,404.56 Da (SMR10), with an average of 13,560.16583 Da. Their isoelectric points varied from 5.80 pH (ZmSMR2) to 10.52 pH (ZmSMR7). Subcellular locations as predicted by Softberry-ProtComp Version 9.0 indicated that ZmSMR4/5/6 were present in the nucleus, with scores of 7.6, 7.5, and 8.0, respectively (Table 1). In contrast, the predicted position of ZmSMR9 was located in the plasma membrane (score of 8.8). The other SMR members showed extracellular localization with relatively low scores. In addition, ZmSMR4/5/6 contained 2–4 skn-1_motifs, the decisive elements for endosperm development [31], which were not found in the sequence of ZmSMR9 (Additional file 3).

**Table 1:** List of identified *SMR* genes in *Zea mays* L. along with their corresponding proteins’ information

Gene	Gene accession. No.	Chromosome No.	CDS length (bp)	Protein length(aa)	Molecular weight(Da)^a^	Isoelectric point(ph)^a^	Sub-cell location (scores)^a^
*ZmSMR1*	Zm00001d042902	chr3	273	90	9272.63	9.10	Extracellular (2.6)
*ZmSMR2*	Zm00001d012956	chr5	288	95	10,211.6	5.80	Extracellular (2.7)
*ZmSMR3*	Zm00001d046896	chr9	426	141	14,441.9	9.76	Extracellular (2.4)
*ZmSMR4*	Zm00001d047159	chr9	381	126	13,850.9	9.83	Nuclear(7.6)
*ZmSMR5*	Zm00001d048533	chr9	411	136	14,773.9	9.33	Nuclear(7.5)
*ZmSMR6*	Zm00001d029820	chr1	411	136	14,719.9	9.66	Nuclear(8.0)
*ZmSMR7*	Zm00001d049769	chr4	321	106	11,526.1	10.52	Extracellular (2.5)
*ZmSMR8*	Zm00001d036683	chr6	456	151	15,494.8	8.92	Extracellular (2.5)
*ZmSMR9*	Zm00001d031546	chr1	381	126	13,246.0	10.25	Plasma membrane(8.8)
*ZmSMR10*	Zm00001d015140	chr5	456	151	16,404.56	9.00	Extracellular (2.4)
*ZmSMR11*	Zm00001d039827	chr3	459	152	15,689.8	10.51	Extracellular (2.3)
*ZmSMR12*	Zm00001d006060	chr2	384	127	13,089.9	10.34	Extracellular (2.5)

^a^The data come from http://www.expasy.org and http://linux1.softberry.com

### Regulatory elements in the promoter sequences of *ZmSMRs*

Cis-elements in the promoter regions are required for temporal, spatial, and cell-specific control of gene expression [32]. The upstream sequences (1.5 kb) of the 12 *ZmSMRs* were submitted to the PlantCARE database, PlantPan 2.0 and RegSite Database of Plant Regulatory Elements-Softberry to identify cis-acting elements. Additional files 2 and 3 list 47 endoreduplication-related elements (E2F elements) [33] situated within the promoter regions of all of *ZmSMRs* except for *ZmSMR6*. Fifteen 5’UTR Py-rich stretch elements, which positively influence the overall expression level [34], were present in the promoter regions of eight *ZmSMR* genes. Eleven promoters contained 1–6 Skn-1_motifs involved in endosperm development [31]. As for salt- or drought stress-related elements, the MBSI motif was predicted only in the promoter of *ZmSMR4*, and nine promoters contained MBS [35]. The low-temperature-responsive element (LTR) [36] and heat-shock element (HSE) [37] were found in six and four genes, respectively, and only the *ZmSMR4* and *ZmSMR5* had both elements (Additional file 3). Two ABA-responsive elements, ABREs and CE3 [38], were detected in twelve and three *ZmSMR* genes, respectively.

### Chromosomal distribution and gene duplication of the SMR gene family in multiple species

**Fig. 1:**
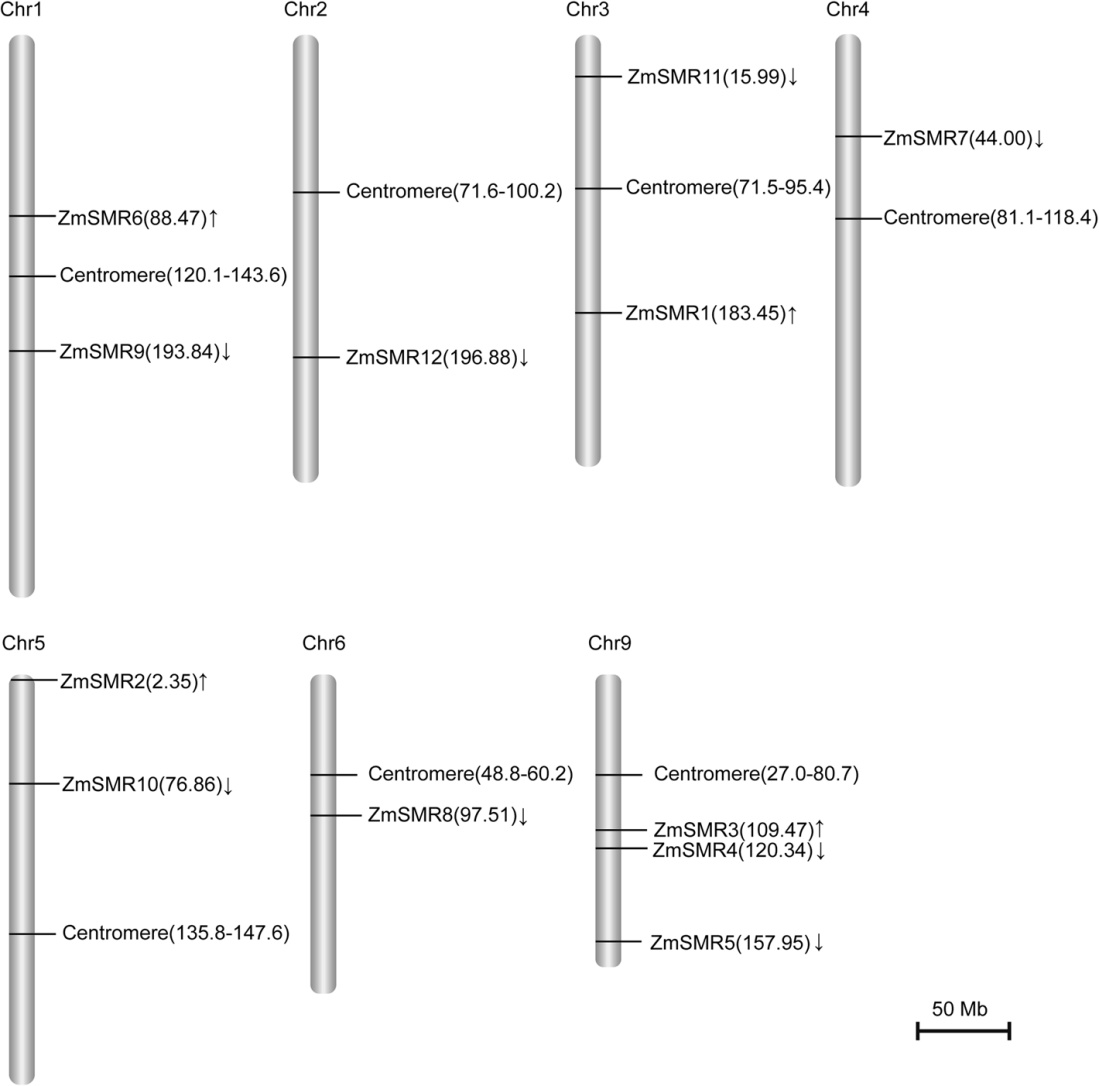
Chromosomal distribution of 12 *ZmSMR* genes. The chromosome number is indicated on the top of each chromosome. The value of the position of each gene and centromere on the chromosome is labeled on the right side of the chromosome bar. The scale of the chromosome length is 50 px = 50 Mb

The physical map positions of the 12 *ZmSMRs* on ten maize chromosomes were identified by BLAST against the maize genome. The 12 genes were distributed non-randomly across 7 out of the 10 maize chromosomes, except for chromosomes 7, 8, and 10 (Fig. 1). Chromosome 9 contained several *ZmSMRs* comprising three members of *ZmSMR3/4/5*, whereas chromosomes 1, 3, and 5 each harbored two *ZmSMR* genes, and chromosomes 2, 4, and 6 carried only one member, namely *ZmSMR12/7/8*.

**Fig. 2:**
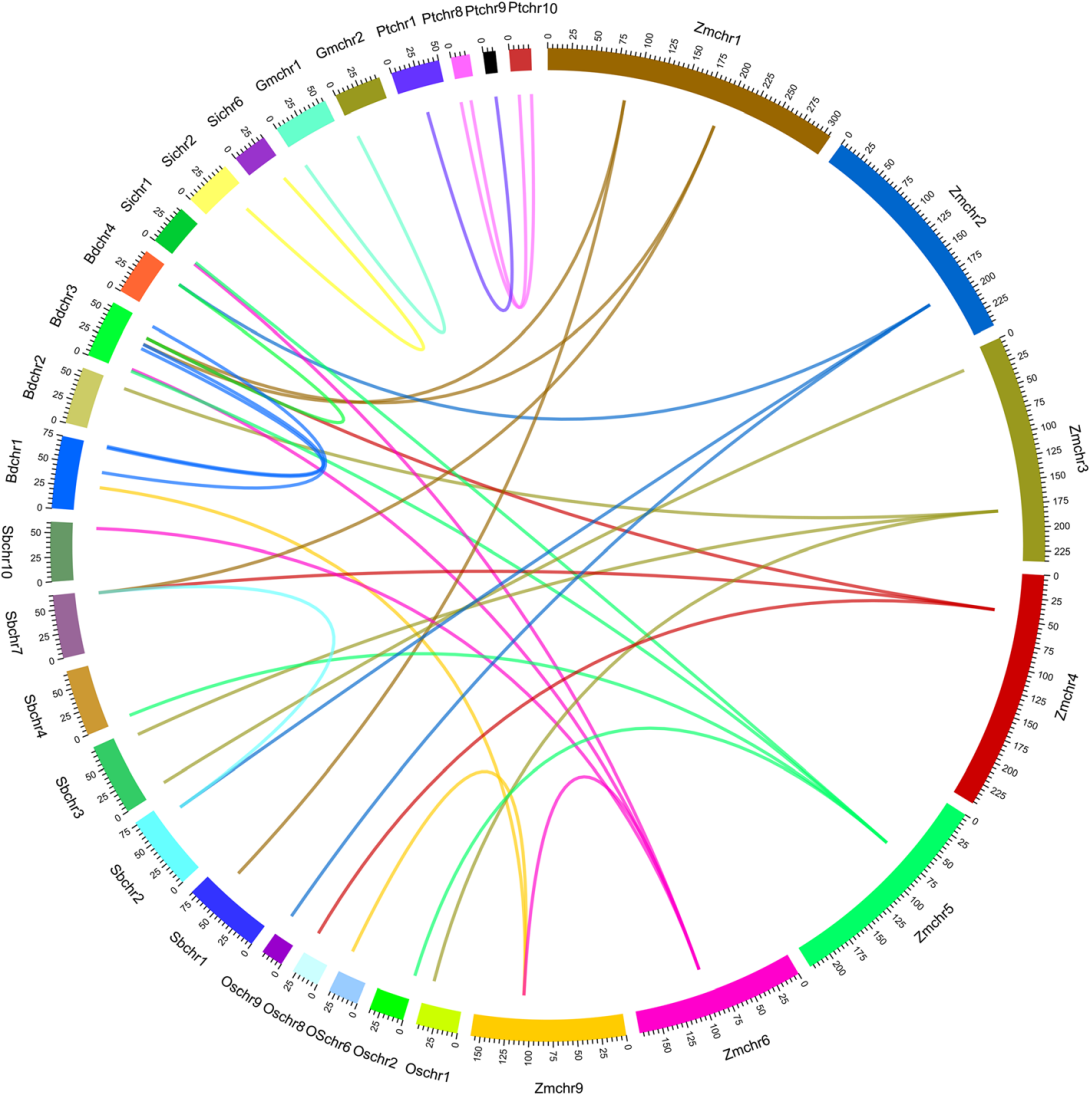
Distribution of genome syntenic and intergenomic synteny *SMR* gene pairs in multiple species. Analyzed species are *Zea mays* (Zmchr), *Brachypodium distachyon* (Bdchr), *Oryza sativa* (Oschr), *Sorghum bicolor* (Sbchr), *Setaria italic* (Sichr), *Populus trichocarpa* (Ptchr) and *Glycine max* (Gmchr). The scale line of the chromosome size is also identified in the figure

Segmental- or whole-genome duplications are common phenomena in plant genome evolution, resulting in the expansion and diversification of many gene families and the evolution of the organism [39]. The maize genome has undergone two rounds of genome duplication, where its size has expanded dramatically (to 2.3 gigabases) over the last three million years via a proliferation of long-terminal-repeat retrotransposons [40]. Among *ZmSMR* genes, only *ZmSMR3* and *ZmSMR8* were found to be a pair of syntenic genes; these were located on chromosomes 9 and 6, respectively (Fig. 2 and Additional file 4). Similarly, only two *SMR* genes were found to be syntenic genes in *Glycine max*, *Sorghum bicolor* and *Setaria italica*. In contrast, there were six (55%) and nine (47%) syntenic genes in *Populus trichocarpa* and *Brachypodium distachyon*, respectively. It is worth noting that there were 23 pairs of syntenic genes in maize and *B. distachyon*, *O. sativa*, *S. bicolor,* and *S. italica* (Fig. 2 and Additional file 4).

### Phylogenetic and conserved-motif analysis of ZmSMRs

**Fig. 3:**
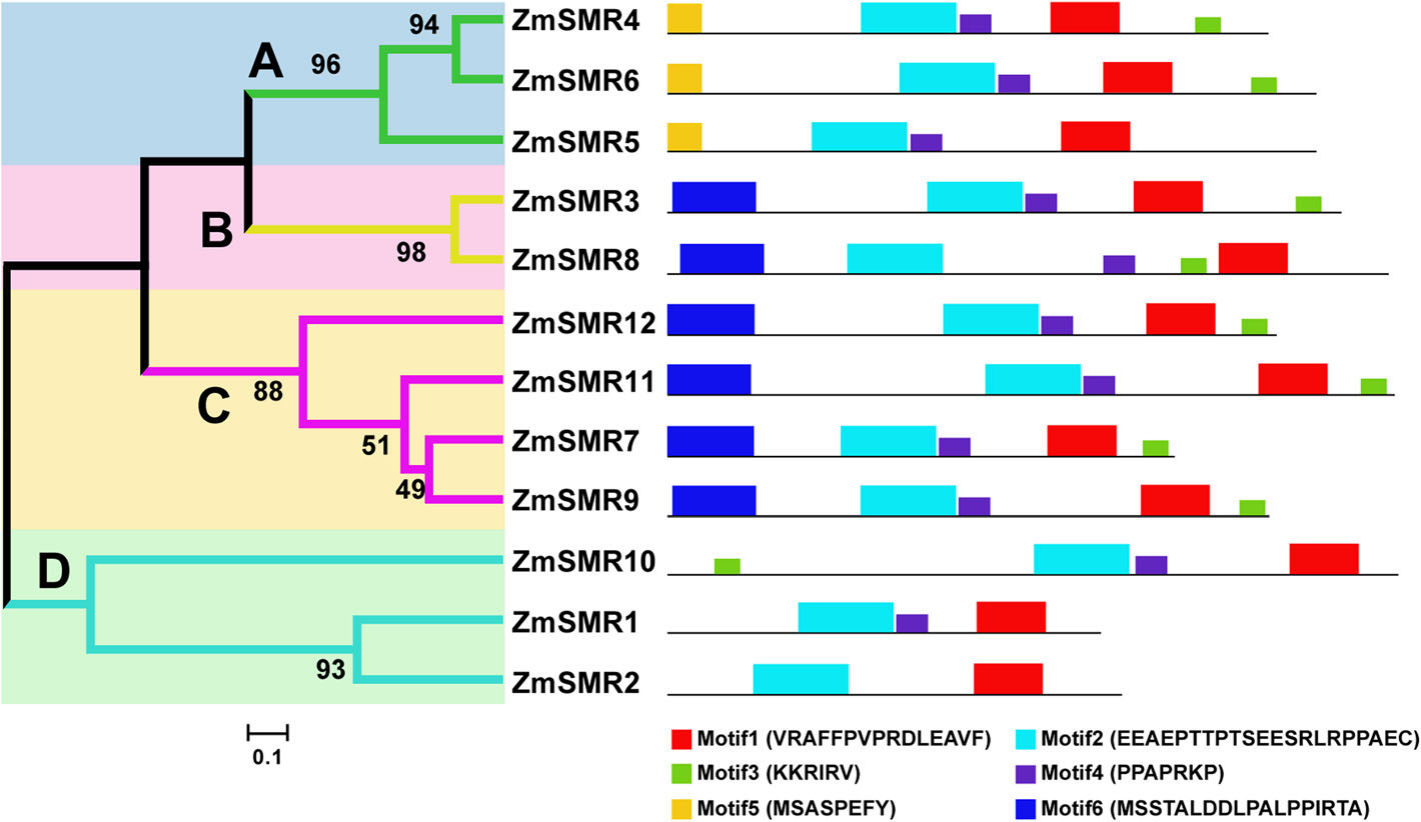
Phylogenetic analysis and motif composition of ZmSMR proteins. The phylogenetic tree was constructed using MEGA6 software by the Neighbor-joining method with 1000 bootstrap iterations based on the 12 full-length amino acids of ZmSMR, and the proteins were clustered into four subgroups (A, B, C, D) marked with different background to facilitate subfamily identification. Schematic representation of the conserved motifs in the ZmSMR proteins was analyzed by MEME. Each motif is represented by a colored box numbered at the bottom, and the consensus sequences of each motif are also shown. The details of sequence logo of the motifs are given in Additional file 6

Phylogenetic reconstruction indicated that the 12 ZmSMRs could be clustered into four distinct subgroups, named A to D (Fig. 3). Subgroup C contained the maximum number (four members) of ZmSMRs, ZmSMR7/9/11/12, followed by subgroups A and D with three members each, and subgroup B with two genes. Furthermore, the ZmSMR3 and ZmSMR8 belonged to subgroup B showed bootstrap values of 98, with alignment identity of 74.05% (Additional file 5), and they were a pair of synteny genes (Fig. 2). For ZmSMR4/6/5, which were predicted to be located in the nucleus (Table 1), members of subgroup A were generally similar in terms of protein length, molecular weight, and isoelectric point.

Six conserved motifs were detected in the 12 ZmSMRs by MEME online search software (Fig. 3b and Additional file 6). The ZmSMRs within the same subgroup possess similar motif compositions. For example, members of subgroup C contained exactly the same motif composition of Motif6-Motif2-Motif4-Motif1-Motif3. Subgroup A harbored motif 5 at its N-terminal, whereas motif 6 was only observed in subgroups B and C (Fig. 3). The results revealed that all members shared motifs 1/2/3/4, except for ZmSMR2 that only harbored motifs 1 and 2. In addition, motifs 1, 2, 4, and 5 were identified by Churchman [30] and are herein described as motifs 2, 4, 1, and 3, respectively. Motif 1 of the ZmSMR family was a domain of the rice EL2 protein that interacts with cyclin [41] and is similar to motif 3 of the CDK-inhibitory ICK/KRP proteins (Fig. 3) [42]. Motif 2 contained a minimum site TP that is phosphorylated by CDKs [43]. This pair of amino acid residues was the most conserved during the evolution of ZmSMR proteins. Motif 4 is rich in proline and consists of a typical PXXP structure followed by one or several amino acid residues. This domain is a protein-interaction site that allows proteins to interact with certain ligands by forming PPII helices [44].

To further examine the evolutionary relationships among the SMR proteins from different species, an unrooted phylogenetic tree was constructed using the full-length SMR proteins from monocotyledons of *Z. mays*, *Oryza sativa*, *S. bicolor*, *S. italica*, *B. distachyon*, the dicotyledon of *A. thaliana*, *G. max*, *P. trichocarpa*, and the musci of *P. patens*. All SMRs from the above species were divided into seven subgroups. No SMR homologous genes were detected in the algae. However, a total of ten SMRs were detected in *P. patens*, nine of which were clustered in Group VI, and the other members of this subgroup were GmSMR1, SiSMR3, PtSMR8, BdSMR17 and OsSMR6. Thus, these five proteins may be very conserved during the evolution of their respective species, and that the ancestors of the SMR family first appeared in the *P. patens.*

**Fig. 4:**
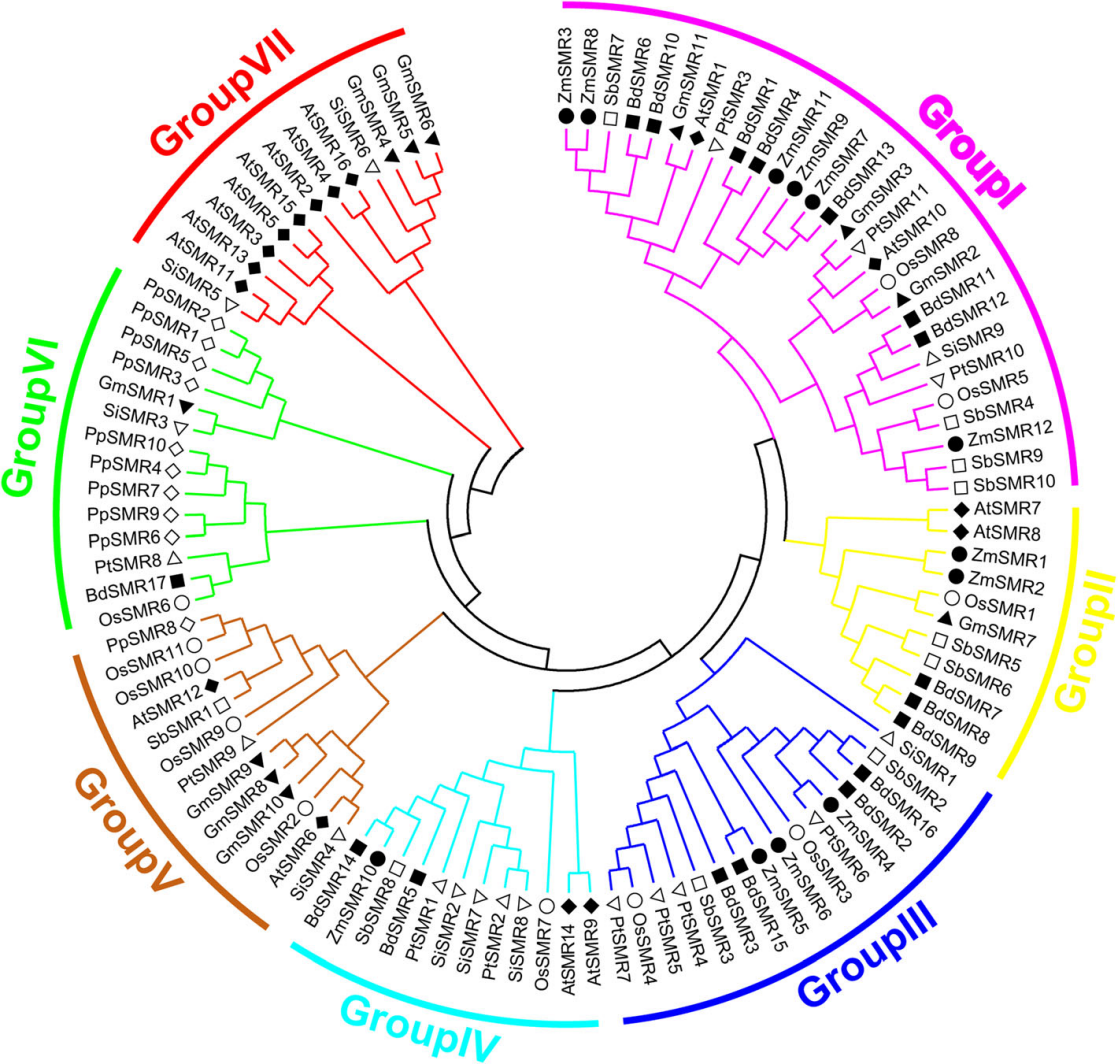
Phylogenetic tree of SMR proteins from *Z. mays*, *Arabidopsis*, *O. sativa* and other species. The complete amino acid sequences of 12 *Z. mays*, 28 *Arabidopsis*, 17 *O. sativa* and 71 SMR proteins from other species were aligned by ClustalW, and the phylogenetic tree was constructed using Molecular Evolutionary Genetics Analysis 6 (MEGA6.06) by the Neighbor-joining method with 1000 bootstrap replicates. Each species’ gene is labeled with a different symbol. ZmSMR represents *Z. mays* SMR; AtSMR represents *A. thaliana* SMR; OsSMR represents *O. sativa* SMR; BdSMR represents *B. distachyod* SMR; SiSMR represents *S. italica* SMR; SbSMR represents *Sorghum bicolor* SMR; GmSMR represents *Glycine max* SMR; PtSMR represents *Populus trichocarpa* SMR; PpSMR represent *Physcomitrella patens* SMR. The phylogenetic tree was divided into six phylogenetic groups as Group I-VII using different colors

All subgroups contained monocotyledons or dicotyledon species except for Group VI (Fig. 4). In addition, the members of the four subgroups, A, B, C, and D were distributed among Groups III/I/II. In total, 18 pairs of SMRs from different species were clustered as pairs (Fig. 4). The proteins of ZmSMR7 and BdSMR13, ZmSMR10 and BdSMR14, ZmSMR5, and BdSMR15 were highly similar, indicating that some consensus in proteins may have existed before the divergence of *B. distachyon* and *Z. mays*. It was worth noting that OsSMR11 and the PpSMR8 were clustered into one pair of genes, indicating that OsSMR11 was very conserved during rice evolution.

### Relationship between grain-filling rate and expression of *ZmSMRs* during maize endosperm development

**Fig. 5:**
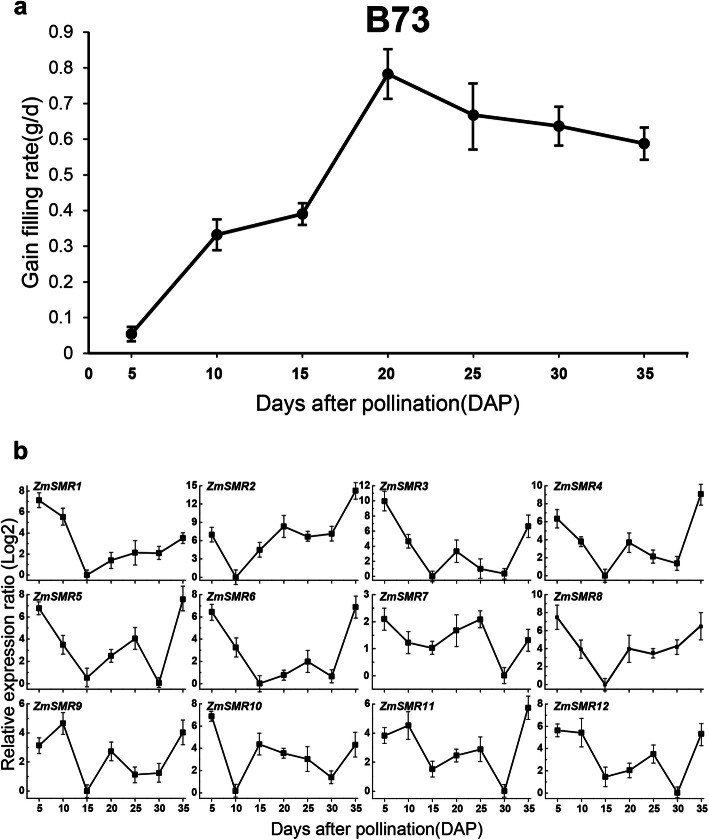
Grain-filling rate and expression profile of the *ZmSMR* family genes in maize endosperm. **a** The grain-filling rate was measured from 5 to 35 DAP every 5 days. The trendline was drawn through the weight increase of hundred-grain weight every 5 days. **b** The maize endosperm samples were taken every 5 days from 5 to 35 DAP. Relative expression ratios in these sample were calculated with reference to the sample in which the respective transcript exhibited the lowest expression. The relative expression values were log2 transformed. The qRT-PCR data were normalized against the expression of Actin as an internal control. Error bars indicate standard deviations. The names of the genes are written in the upper right corner of each bar diagram

Maize embryo tissue is relatively young, and endoreduplication activity often occurs in this rapidly developing and highly metabolically active tissue [6]. To test the role of *ZmSMRs* in the development of maize embryos, we examined the grain-filling rate of the inbred line, B73, every 5 days from 5 to 35 DAP. The grain-filling rate increased sharply at 5–10 DAP and 15–20 DAP, coinciding with the beginning and active periods of endoreduplication, respectively. In contrast, a moderate trend in grain-filling rate was observed from 10 DAP to 15 DAP when endoreduplication activity was initiated (Additional file 10). Endoreduplication levels were lower during the programmed cell death (PCD) stage, which was observed at 20 DAP [45], occurring simultaneously with the gradual reduction in the grain-filling rate (Fig. 5a).

Gene expression may provide a value clue for exploring gene function [46]. Here, quantitative real-time PCR (qRT-PCR) was used to analyze the expression profiles of the 12 *ZmSMRs* in the endosperm every 5 days from 5 to 35 DAP (Fig. 5b). The univariate linear regression statistics of *ZmSMR* gene expression and grain filling rate showed that the expression level of almost all genes was negatively correlated with the rate of grain filling, with *ZmSMR1/12* reaching an extremely significant level (*P* < 0.01) and *ZmSMR6* reaching a significant level (*P* < 0.05). Multiple linear regression showed a correlation coefficient of 0.977, which was extremely significant (*P* < 0.01) (Table 2). The 12 *ZmSMR* genes were significantly upregulated at 5 DAP, 20 DAP and 25 DAP, coinciding with a dramatic increase in grain filling rate. These genes have both motif 1 and motif 2. Motif 1 may be the CDK-inhibitory ICK/KRP protein (Fig. 3) [42]. The TP site of Motif 2 is involved in CDK regulation of the cell cycle [43].

**Table 2:** Regression statistics of Grain filling rate and ZmSMR gene expression: univariate and multivariate linear regression

Dependent variable	Independent variable–univariate regression (r)^a^												Multivariate Ra^b^
	ZmSMR1	ZmSMR2	ZmSMR3	ZmSMR4	ZmSMR5	ZmSMR6	ZmSMR7	ZmSMR8	ZmSMR9	ZmSMR10	ZmSMR11	ZmSMR12	
Grain filling rate	0.698(−)	0.349	0.139(−)	0.189(−)	0.34(−)	0.522(−)	0.196(−)	0.267(−)	0.2(−)	0.374(−)	0.267(−)	0.55(−)	0.977
Regression coefficient significance	0.001	0.121	0.547	0.411	0.132	0.015	0.393	0.243	0.384	0.095	0.242	0.010	0.001

(−) indicates negative linear relationship

^a^r indicates the coefficient of determination of the independent variable, the significance was checked by t-test

^b^Ra = adjusted r of the multiple linear regression model and the significant probability from the ANOVA table in the multiple linear regression model

The expression patterns of genes can provide a preliminary indication for revealing its function [47]. Expression profiling of 12 *ZmSMR* genes in maize endosperm from 5 to 35 DAP was performed. In general, the expression pattern of *ZmSMR* genes exhibited a “W” shape, wherein they were upregulated at 5 DAP, 20 DAP, and 25 DAP, 35DAP, which coincided with the rapid increase in the grain-filling rate that in turn contributed to the development of the maize endosperm.

*ZmSMR9/11/12* was upregulated at 10 DAP, whereas *ZmSMR2/10* was highly expressed at 15 DAP. These two groups of genes exhibited similar expression patterns, and they belonged to subgroups C and D, respectively. The endoreduplication had already started in the endosperm of 5 DAP due to the appearance of 12 C. The highest ploidy level was reached at 20–30 DAP, maintaining 96 C (Additional file 10). Our results indicated that ZmSMRs, although negatively correlated with grain filling, may promote endoreduplication during the 5, 20, and 25 DAP phases.

### Expression patterns of *ZmSMRs* in diverse tissues of maize

**Fig. 6:**
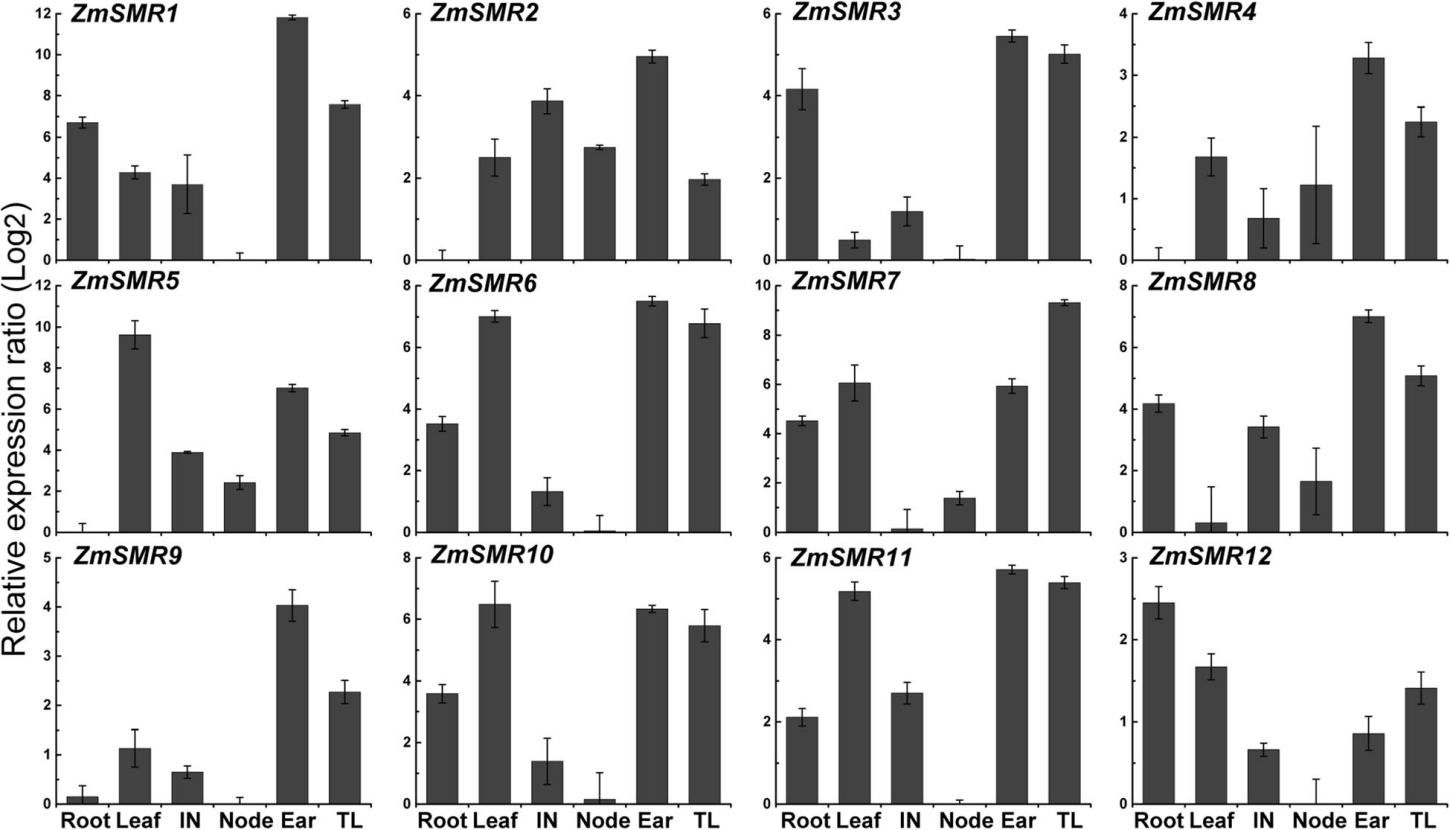
Expression analysis of 12 *ZmSMR* genes in different tissues. Relative quantities of 12 *ZmSMR* members in Root, Leaf, Internode (IN), Node, Ear and Tassel (TL) tissues of maize determined using qRT-PCR. Relative quantities in different tissue samples were calculated with reference to tissue samples in which the respective transcript exhibited the lowest expression. The relative expression values were log2 transformed. The qRT-PCR data were normalized against the expression of Actin as an internal control. Error bars indicate standard deviations. Gene-specific primers were used for qRT-PCR analysis of *ZmSMR* genes

Transcript levels of all *ZmSMRs* were examined in diverse maize tissues, including root, leaf, internode, node, ear, and tassel (Fig. 6). High expression levels of all *ZmSMRs* were observed in the ears and tassels. Therein, eight genes (*ZmSMR1*, *ZmSMR2*, *ZmSMR3*, *ZmSMR4*, *ZmSMR6*, *ZmSMR8*, *ZmSMR9,* and *ZmSMR11*) were expressed the highest in ears; one gene (*ZmSMR7*) had its strongest expression in tassels. *ZmSMR12* were expressed predominantly in roots, and *ZmSMR5/10* occurred predominantly in leaves. The mRNA accumulation levels of *ZmSMR6/10/11* in leaves, ears, and tassels were similar. Most genes, especially the *ZmSMR1/3/6/9/10/11/12*, were expressed relatively lower, in nodes and internodes. The expression levels of *ZmSMR8* and *ZmSMR2/4/5* were barely detected in leaves and roots, respectively. *ZmSMR3* and *ZmSMR8*, which were clustered based on protein motif analysis (Fig. 3), shared a similar expression pattern. The tissue-specific expression patterns of *ZmSMRs* indicate that endoreduplication commonly occurs in various maize tissues, especially in the ears, tassels, and leaves.

### Expression profiles of *ZmSMRs* in response to abiotic stresses

**Fig. 7:**
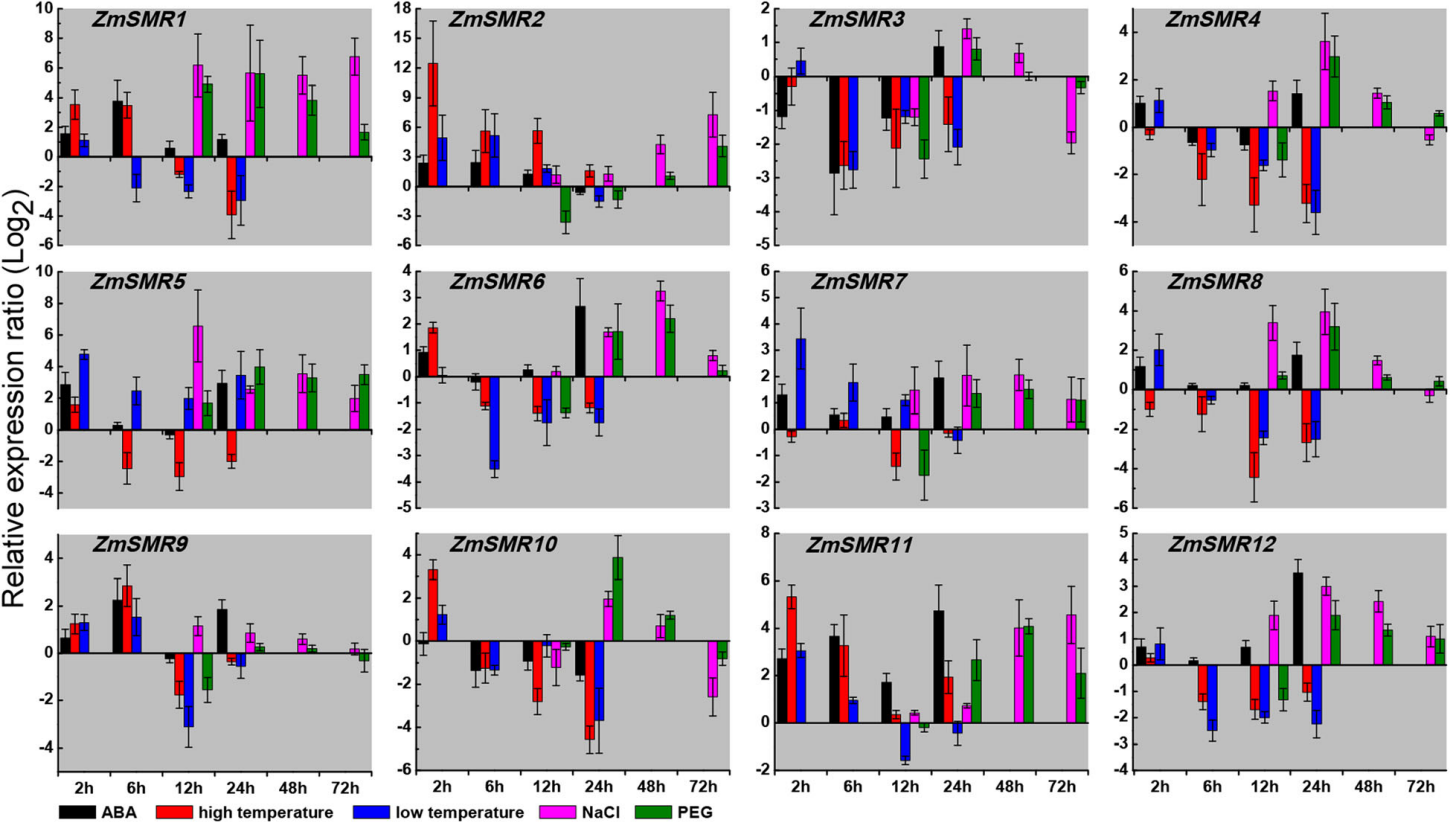
Expression profiles of *ZmSMR* genes under various abiotic conditions. The x-axes represent treatment time, and y-axes indicate scales of relative expression levels. Three-leaf maize seedlings were treated with 4 °C (low temperature), 42 °C (high temperature), or 100 μM of ABA for 24 h, or with 20% PEG and 200 mM of NaCl for 72 h. Mixed leaves of three seedlings as well as the untreated leaves were collected after treatment for 2, 6, 12, 24, 48, 72 h, where the untreated leaves were regarded as the control check (CK). The relative expression values were log2 transformed. The qRT-PCR data were normalized against the expression of Actin as an internal control. Error bars indicate standard deviations

Endoreplication is affected by the environment and by plant hormones and has been reviewed by Barow [15]. The *ZmSMRs* contain a variety of cis-acting elements, and these are all expressed in the leaves. Therefore, we investigated the responses of these genes in the leaves under multiple abiotic stress conditions, including ABA (100 μM), heat (42 °C), cold (4 °C), salt (200 mM NaCl), and drought (20% PEG) (Fig. 7). The qRT-PCR indicated that the increased mRNA levels of *ZmSMR3*, *ZmSMR11*, and *ZmSMR12* were induced by ABA stress in a time-dependent manner. However, *ZmSMR4*, *ZmSMR5*, *ZmSMR6*, *ZmSMR7*, and *ZmSMR8* were significantly upregulated at the 2-h and 24-h time points and were downregulated at the 6-h and 12-h time points (Fig. 7). For the heat and cold treatments, *ZmSMR5*, *ZmSMR7*, and *ZmSMR11* were differentially expressed at specific time points. In contrast, the expression patterns of the other genes were highly similar. *ZmSMR3*, *ZmSMR4*, and *ZmSMR12* were downregulated at all time points. However, *ZmSMR2* was upregulated after the application of heat and cold stress. For salt and drought stress, *ZmSMR4* and *ZmSMR8* were upregulated from 12 h to 48 h after stress application, and then were downregulated at 72 h after exposure to stress. *ZmSMR1* was significantly upregulated at all time points during stress application. The responses of all *ZmSMR* genes to salt and drought were highly similar at all four time points (Fig. 7). This conformity may be due to cis-acting elements that are involved in salt and drought stresses; both MBSI and MBS [35]. These results indicate that the *ZmSMR* genes might be involved in stress response to adverse environmental conditions.

## Discussion

The *SIM* was first isolated in *A. thaliana* and was determined to have dual functions as a suppressor of mitosis and an elicitor for endoreplication [29]. In addition, the SIM is the only plant factor that has been reported to date that promotes the transformation from mitosis to endoreplication. The tissue-specific expression pattern of the *SIM* has been studied in *A. thaliana*, and a several bioinformatic analyses of the *SMR* family have been reported [30], whereas *SMR* genes have not been well studied in maize.

In the present study, 12 *ZmSMR* genes were identified in the maize genome via a homology-search method using *Arabidopsis* SIM/SMR aa sequences as the query [48]. These 12 *ZmSMR* genes showed significantly different isoelectric points and marked variations in their predicted sub-cellular locations (e.g., *ZmSMR4/5/6* was predicted to be situated in the nucleus with a high prediction score, Table 1). Moreover, the 12 *ZmSMR* genes were nonuniformly distributed across 7 out of the 10 chromosomes of maize (Fig. 1). The observed differences in the characters of the *ZmSMR* family members and *ZmSMR4/5/6* may reflect their distinct roles in initiating endoreplication.

In previous studies, three motifs were identified in the *Arabidopsis* SMR family [43], whereas in another study, five motifs were described by aligning the putative protein product of the SIM reading frame and related plant proteins from *S. lycopersicum*, *S. tuberosum*, *Z. mays*, *O. sativa*, *P. tremula*, and *G. max* [30]. However, in this study, six motifs were identified in the ZmSMRs. The order of these motifs on the ZmSMR protein sequences was basically the same, except for ZmSMR10, in which motif 3 was located at the N-terminal. It was worth noting that only *ZmSMR10* was upregulated at 15 DAP, indicating that the order of motifs may also affect the transcription of the entire gene. However, ZmSMR1 and ZmSMR2 did not contain motif 3. The phylogenetic analysis showed that ZmSMR1/2/10 belong to the same subgroup (Fig. 3), thereby suggesting similar functions, and that a change in the position of motif 3 within the polypeptide may lead to a loss of function. Furthermore, the two new motifs 5 and 6 were detected only in maize. Both were located at the N-terminal and contained aa residues with MS in order. Motif 5 (MSASPEFY) was also detected consistently as domain 12 in the EL2 protein in rice, which was a plant-specific cyclin dependent kinase inhibitor [41], suggesting that motif 5 in ZmSMR4/5/6 may play an important role in the transformation from mitosis into endoreplication. The expression patterns of *ZmSMR* genes in endosperm showed they were upregulated at 5 DAP, 20 DAP, and 25 DAP, 35 DAP, thereby implying their importance during the initiation and progression of endoreproduction. Studies have shown that the nucleus size from the central endosperm to the aleurone layer of maize gradually decreases, indicating that the internal replication process develops from the endosperm center to the edge along a gradient [4]. The expression of *ZmSMRs* is time-specific, which may be due to expression in different endosperm subaleurone domains at different stages of endosperm tissue development. The endosperm cells in the center of 8–10 DAP grains gradually shifted from mitosis to internal replication cycles [4, 18]. In addition, all *ZmSMRs* were highly expressed during 5–10 DAP; *ZmSMR9/11/12* was especially up-regulated during this period, indicating that these three genes may play a major role in initiating endoreplication of endosperm central cells. At 16 DAP, most cells in the endosperm entered endoreplication [49]. At the same time, the mitotic index of the endosperm had fallen to less than 1%. The qRT-PCR results showed that ZmSMR2/10 were up-regulated at 15 DAP, indicating that they may be mainly responsible for the endoreplication regulation of outer endosperm or aleurone cells. It has been reported that expression of CYCB1; 1 and CYCB1; 2 are reactivated in the trichome of *SIMESE*-mutated *Arabidopsis* [30]. In the developing maize endosperm, the expression of CDKA protein is relatively stable during the cell cycle, while the expression of CDKB associated with the M phase is decreased [21]. Therefore, ZmSMRs as CKIs may inhibit the expression of CYCB1;1 and CYCB1;2 by degrading CDKB and thereby maintain the cells in a state of endoreplication. Some maize endosperm cells enter the PCD stage at 20 DAP [45]; however, endoreduplication can mark cells for programmed death [19]. At 35 DAP, all *ZmSMRs* were upregulated. These genes may thus possibly regulate the transition of endosperm cells from the endoreplication stage to the apoptosis stage, thereby reducing the grain filling speed. This may also be due to the gradual drying of the endosperm during this period, which causes the up-regulation of *ZmSMRs* expression in ABRE-rich elements. However, further details concerning the mechanisms underlying maize endoreplication require additional investigation.

*SIM* was first detected in the trichomes of *A. thaliana*, and it has also been studied in the roots, rosettes, stems, siliques, and flowers and is particularly upregulated in the roots and stems [30]. Analysis of tissue expression indicated that all *ZmSMR* genes were upregulated in the ears and tassels and thus may be involved in regulating the development of reproductive organs. In contrast, *ZmSMR2/4/5/9* were downregulated in the roots, which may be due to the differentiation of their functions compared to the *SIM* gene. *SMR3* and *SMR8* belonged to Group B and showed similar expression profiles, suggesting that their functions may be highly similar.

Endoreplication is not only related to plant growth and development [6, 8, 11, 12] but also to various abiotic stresses [13, 14]. Moreover, almost all the promoter regions of *ZmSMRs* have the ABRE cis-elements in response to ABA stress [38] and E2F cis-elements that regulate endoreplication [33]. In this study, five stress conditions were examined (ABA, heat, cold, salt, and drought). The observed upregulation of ZmSMR genes was apparently in response to at least one of the stress conditions. ABA has recently been reported to play crucial roles in response to abiotic stresses, such as drought or salinity [50] that cause the G1-to-S transition to be impaired, thereby slowing DNA replication and/or delaying entry into mitosis [41]. CKI is a candidate protein involved the molecular mechanisms that link the stress perception directly to the cell-cycle machinery [41]. SMR, as a kind of CKI, may also be associated with response to environmental stresses. In this study, *ZmSMR9*, *ZmSMR11* and *ZmSMR12* genes were induced under ABA stress, and they may specifically express in the center of the endosperm tissue, indicating that these three genes may play direct or indirect roles in response to ABA stress and in seed development. Phylogenetic analysis also showed that they all belonged to subgroup C, indicating that their functions were more conservative. In addition, the tendency of *ZmSMRs* to be up- or down-regulated under hot and cold stress was very similar to ABA stress, indicating that induction of *ZmSMRs* by hot and cold occurred in an ABA-dependent manner. The expression of *ZmSMR5* and *ZmSMR7* under cold treatment had been induced and was the same as the response of the rice EL2 gene to cold stress [41]; EL2 was identified as a cell-cycle regulatory gene related to the *SIM* gene of *Arabidopsis thaliana.* These results show that overexpression of ZmSMR5/7 might be responsible for cold tolerance. Endoreplication can also allow cells to adapt to salt stress [13], and Churchman confirmed this conclusion by reporting that *Arabidopsis SMR3/4/5* was up-regulated by salt stress [41]. In addition, drought can inhibit endoreplication in maize endosperm [51, 52]. The rice *EL2* was also induced after 24 h of drought treatment [41]. Similarly, *ZmSMR1*, *ZmSMR5, ZmSMR11* and *ZmSMR12* were upregulated by salt and drought stresses, indicating that they may play important roles in enhancing tolerance to salt and drought stresses in maize seeding development. In general, the functions of *ZmSMR* genes are diverse and are strongly associated with response to abiotic conditions, although the underlying regulatory mechanisms may be complex. A follow-up study on how to utilize these functions in improving resistance in maize may be necessary.

In summary, the distribution, structure, and phylogenetic relationships of members of the ZmSMR protein family were comprehensively analyzed in the present study. In addition, these characteristics of *ZmSMR* genes were correlated with the development of maize endosperm and tissue expression in response to abiotic stresses. These results may improve our understanding of the role of the *ZmSMR* family in maize and should lay the foundation for future research.

## Conclusions

Twelve putative ZmSMRs were identified by comparison of the SIM/SMR aa sequence in *Arabidopsis* to the maize genome. The genes were divided into four groups via phylogenetic analysis. Conserved domains consisted of six motifs that were predicted and that supported the clustering results. Four motifs were reported to be associated with the regulation of replication. The temporal expression characteristics of the endosperm development of maize seeds indicate that the *ZmSMR* gene is negatively correlated with grain filling but that it can initiate the start of endoreduplication at 5 DAP and maintain the high ploidy of endosperm cells at 96 C during 20 and 25 DAP. In addition, *ZmSMR9/11/12* may regulate the initiation of endoreplication of endosperm central cells. *ZmSMR2/10* may be mainly responsible for the endoreplication regulation of outer endosperm or aleurone cells. In addition, *ZmSMR9/11/12* were upregulated under ABA stress, indicating that they may regulate endoreduplication and adaptation to abiotic stresses, either directly or indirectly. The *ZmSMR* genes were highly expressed in the ears and tassels, as shown by tissue-specific analysis, indicating that they might be involved in regulating the development of the reproductive organs. These results may provide valuable information for future studies of the function of the *SMR* family in maize.

## Methods

### Identification of *SMR* genes in maize

To identify the *SMR* genes in the B73 reference genome (RefGen_v4), we used the reported *Arabidopsis* SIM/SMR amino acid sequences [48] as queries in a BLASTP search against all maize proteins downloaded from the Phytozome database using an e-value of 1e-5 and identity of 50% as thresholds. The keywords “maize SIM” and “maize SMR” were used as queries to search against the NCBI protein database. The BLASTP and database search hits were compared and parsed by manual editing. Furthermore, a BLASTN of its own genome was performed in maizeGDB to find possible missing gene models. A self-BLAST of these sequences was also performed to remove redundant sequences, and then the remaining sequences were submitted to the NCBI-CDD web server to confirm the presence and integrity of the conserved domains. After manual correcting, the putative ZmSMR proteins were obtained. Then the Expasy ProtParam tool (https://web.expasy.org/protparam/) and Softberry-ProtComp Version 9.0 (http://www.softberry.com/berry.phtml) were used to determine the physicochemical parameters and subcellular localization of the maize SMR proteins, respectively.

### Regulatory elements in the promoter region of *ZmSMRs*

A 1.5-kb DNA sequence upstream of the initiation codon (ATG) of each *ZmSMR* was downloaded from the Phytozome database [53]. The transcription start site was designated as + 1. The elements in the promoter fragments (from − 1500 bp to + 1 bp) of the *ZmSMR* genes were identified using the PlantCARE (http://bioinformatics.psb.ugent.be/webtools/plantcare/html/) [53, 54], PlantPan 2.0 (http://plantpan2.itps.ncku.edu.tw) [55] and RegSite Database of Plant Regulatory Elements - Softberry online program (http://www.softberry.com/berry.phtml) [56, 57].

### Identification of syntenic genes

The syntenic relationships of the maize genes were identified by comparing maize B73 genome sequences using the SynMap [58] utility of the CoGe website (https://genomevolution.org/coge/). The syntenic genes were detected using CDS data with default settings except for the Quota Align Merge algorithm, and the final syntenic gene-set output with GEvo links was downloaded for further analysis [59].

### Phylogenetic analysis and conserved motifs analysis

Multiple sequence alignment of the full-length protein sequences of ZmSMRs was performed using ClustalW as integrated into MEGA 7.0 with default parameters. The phylogenetic reconstruction was done with MEGA 6.0 using the Neighbor-joining method with 1000 bootstrap replicates [60]. To study the phylogenetic relationship of SMR proteins in *Z. mays*, *A. thaliana*, *O. sativa*, *S. bicolor*, *S. italica*, *B. distachyon*, *G. max*, *P. trichocarpa* and *P. patens*, full-length SMR aa sequences were retrieved from the NCBI and Phytozome databases. In addition, a BLAST of its own genome was performed for these acquired genes to find possible missing gene models. Multiple sequence alignment was performed, and an unrooted tree was plotted as described previously. In addition, to further examine the diversity of motif compositions in the putative ZmSMR proteins, multiple expectation maximization for motif elicitation (MEME) online search software [61] was used to predict the conserved motifs in these proteins. The maximum number of motifs was set to six [62].

### Sampling of seeds at different DAP and grain filling rates

The maize (*Z. mays* L.) inbred line B73 was provided by the Key Laboratory of Biology and Genetic Improvement of Maize in Arid Areas of the Northwest Region, Ministry of Agriculture, College of Agronomy, Northwest A&F University. The lines were grown in the field in the summer of 2012 in Yangling, Shaanxi, China. Ears were self-pollinated on the same day. Seed endosperm at the same position in maize ears was collected every 5 days from 0 to 30 DAP (5 DAP for whole seeds), frozen in liquid nitrogen, and then stored in a − 80 °C freezer. RNA was isolated from a portion of the seeds and used in testing the relative expression of *SMR* genes during the development of maize endosperm. The rest of the seeds were kept at 105 °C for half an hour to deactivate and were then dried to a constant weight at 80 °C. The grain-filling rate was calculated by dividing the increment of hundred-grain weight by the number of days and kernels between two adjacent grain filling stages [16]. Three biological replicates were performed in the qRT-PCR analysis.

### Plant growth conditions and stress treatments

The maize inbred line B73 seeds were immersed in 10% H_2_O_2_ for 30 min to disinfect and were then treated with 3% CaSO3 for 3 h to promote germination. Treated seeds were grown in Hoagland’s solution in a greenhouse under a 14-h light and 10-h dark cycle at 23 ± 1 °C. Leaves were collected from maize seedlings at the three-leaf stage and were then used in the expression profiling of *ZmSMR* genes under ABA, heat, cold, salt, and drought stresses. For heat and cold stress, the maize seedlings were placed in 42 °C and 4 °C environments, respectively. The maize seedlings were immersed in a 100 μM ABA solution for hormone treatment. For these three stresses, the leaves from the seedlings were collected after 2, 6, 12, and 24 h. The solutions for salt and drought treatments were prepared by adding NaCl (200 Mm/L) and PEG [20% (m/v)] to full-strength Hoagland’s solution, respectively. Both treatments lasted 12 h, 24 h, 48 h, and 72 h. Control check (CK) seedlings were kept in the unstressed growth conditions. Three biological replicates were performed in the qRT-PCR analysis.

### RNA isolation and quantitative real-time PCR

Total RNA was extracted using the TaKaRa MiniBEST Plant RNA Extraction Kit (Takara, Dalian, China). RNase-free DNase-I was used to remove any contaminating genomic DNA in the solution. First-strand cDNA synthesis was conducted using a FastQuant RT Kit (TIANGEN, Beijing, China) according to the manufacturer’s instructions. The qRT-PCR analysis of all 12 *ZmSMRs* was performed using primers that were designed according to the *ZmSMRs*’ sequences and using an NCBI Primer-BLAST online instrument (Additional file 11). The amplification products were controlled within a size range of 130 bp to 250 bp (Additional file 11). The maize *actin* (Zm00001d013873) gene was used as an internal control. All primers were synthesized by Sangon Biotech Company. The qRT-PCR assays were performed in optical 96-well plates with three technical replicates using the BIO-RAD CFX96 Detection System (Bio-Rad, CA, USA). Each reaction was performed in 20 μL of a SuperReal Premix Plus (SYBR Green) reaction mixture (TIANGEN, Beijing, China), following the manufacturer’s instructions. The relative expression ratio of each gene was calculated using the 2^-△△CT^ method [63].

## Supplementary information

**Additional file 1.** Gene ID and Sequence of ZmSMRs.

**Additional file 2 **The numbers and locations of regulatory cis-acting elements included in *ZmSMR* genes. (a) The number of genes for every cis-acting element. E2Fa/b, endoreduplication starting element (En); 5’UTR Py-rich stretch (5UTR), cis-acting element conferring high transcription levels; Ry-element, cis-acting regulatory element involved in seed-specific regulation; Skn-1_motif, cis-acting regulatory element required for endosperm expression; MBS/ MBSI, MYB binding site involved in drought-inducibility; HSE, cis-acting element involved in heat stress responsiveness; LTR, cis-acting element involved in low-temperature responsiveness; ABRE/CE3, cis-acting element involved in ABA responsiveness. (b) The location of these regulatory cis-acting elements in *ZmSMR* gene promoters. The elements are distinguished by different colors. The upstream sequence scale is shown above, and the consensus sequences of each component are also shown at the bottom of the figure. Detailed locations, functions, and numbers can be found in Additional file 3.

**Additional file 3 **The number of various cis-acting elements in *ZmSMR* promoters.

**Additional file 4.** Genomic and intergenomic synteny SMR gene pairs in multiple species.

**Additional file 5.** Alignment results of genes on the same branch of the ZmSMR genes phylogenetic tree.

**Additional file 6.** Overview of conserved motifs of ZmSMR genes identified through MEME analysis.

**Additional file 7 **List of the SMR protein sequences in maize, *Arabidopsis thaliana*, rice, Brachypodium distachyod, millet, sorghum, soybean and *Populus trichocarpa*.

**Additional file 8 **Number of SMR in maize, *Brachypodium distachyon*, barley, millet, sorghum, soybean, *Populus trichocarpa*, rice, Arabidopsis.

**Additional file 9 **Orthology genes of maize in *Sorghum bicolor*, *Setaria italica*, *Oryza sativa* and *Brachypodium distachyon*.

**Additional file 10.** The number of haploid genomes in different ploidy level at different times after pollination.

**Additional file 11 **Primers for qRT-PCR to investigate the *ZmSMR* genes.

## Data Availability

The datasets supporting the conclusions of this article are included within the article and its additional files.

## Abbreviations

SIM: SIAMESE
CKI: Cell cycle kinase inhibitor
SMR: SIAMESE-RELATED
ZmSMR: *Zea mays* L. SIAMESE-RELATED
DAP: Days after pollination
CDKs: Cyclin-dependent kinases
CYC: Cyclin
CYCDs: D-type cyclins
DE: Decision Element
HSE: Heat shock element
LTR: Low temperature responsive element
ABREs: ABA-responsive elements
