# Supplementary figures and images for: Genome-wide evolutionary characterization and expression analysis of SIAMESE-RELATED family genes in maize
Source: BMC Evol Biol. 2020 Jul 29;20:91. doi: 10.1186/s12862-020-01619-2 (PMC7389639; doi:10.1186/s12862-020-01619-2)

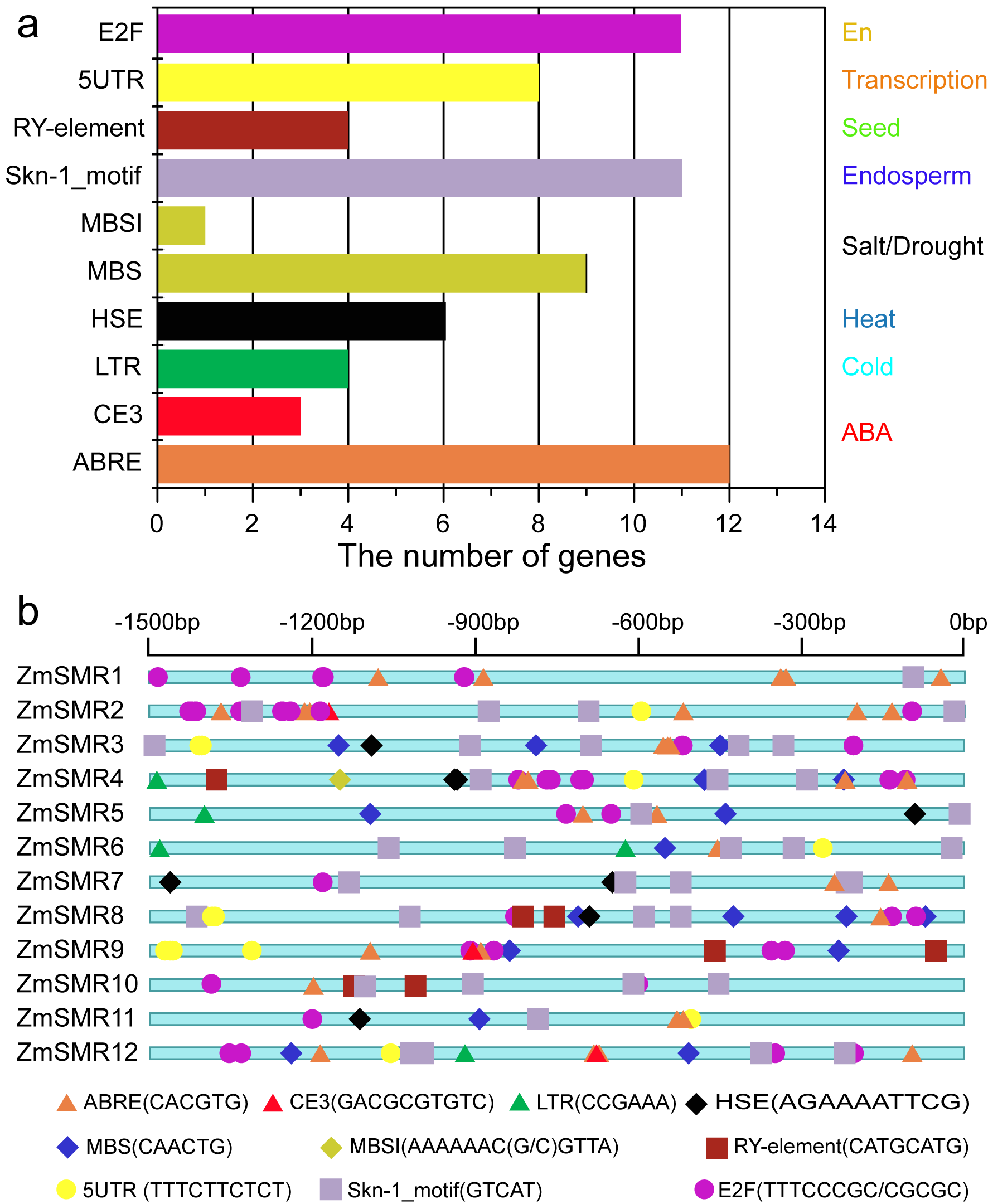

Supplement: Supplementary file 2 — Additional file 2 The numbers and locations of regulatory cis-acting elements included in ZmSMR genes. (a) The number of genes for every cis-acting element. E2Fa/b, endoreduplication starting element (En); 5’UTR Py-rich stretch (5UTR), cis-acting element conferring high transcription levels; Ry-element, cis-acting regulatory element involved in seed-specific regulation; Skn-1_motif, cis-acting regulatory element required for endosperm expression; MBS/ MBSI, MYB binding site involved in drought-inducibility; HSE, cis-acting element involved in heat stress responsiveness; LTR, cis-acting element involved in low-temperature responsiveness; ABRE/CE3, cis-acting element involved in ABA responsiveness. (b) The location of these regulatory cis-acting elements in ZmSMR gene promoters. The elements are distinguished by different colors. The upstream sequence scale is shown above, and the consensus sequences of each component are also shown at the bottom of the figure. Detailed locations, functions, and numbers can be found in Additional file 3. [file 12862_2020_1619_MOESM2_ESM.tif]

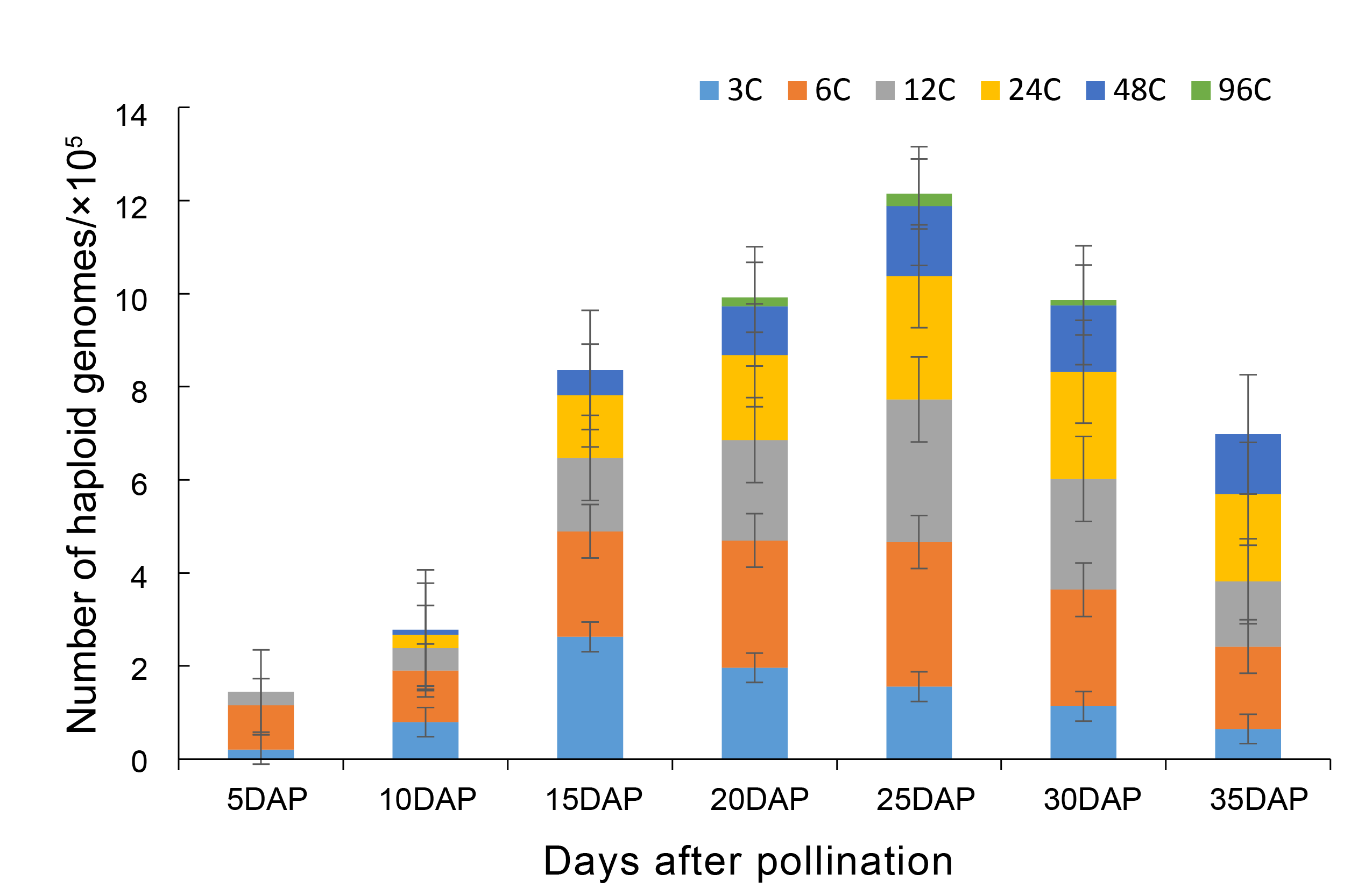

Supplement: Supplementary file 10 — Additional file 10. The number of haploid genomes in different ploidy level at different times after pollination. [file 12862_2020_1619_MOESM10_ESM.tif]
