# Supplementary material for: Genome-wide evolutionary characterization and expression analysis of SIAMESE-RELATED family genes in maize
Source: BMC Evol Biol. 2020 Jul 29;20:91. doi: 10.1186/s12862-020-01619-2 (PMC7389639; doi:10.1186/s12862-020-01619-2)
Supplement: Supplementary file 6 — Additional file 6. Overview of conserved motifs of ZmSMR genes identified through MEME analysis. [file 12862_2020_1619_MOESM6_ESM.ppt]

## Slide 1
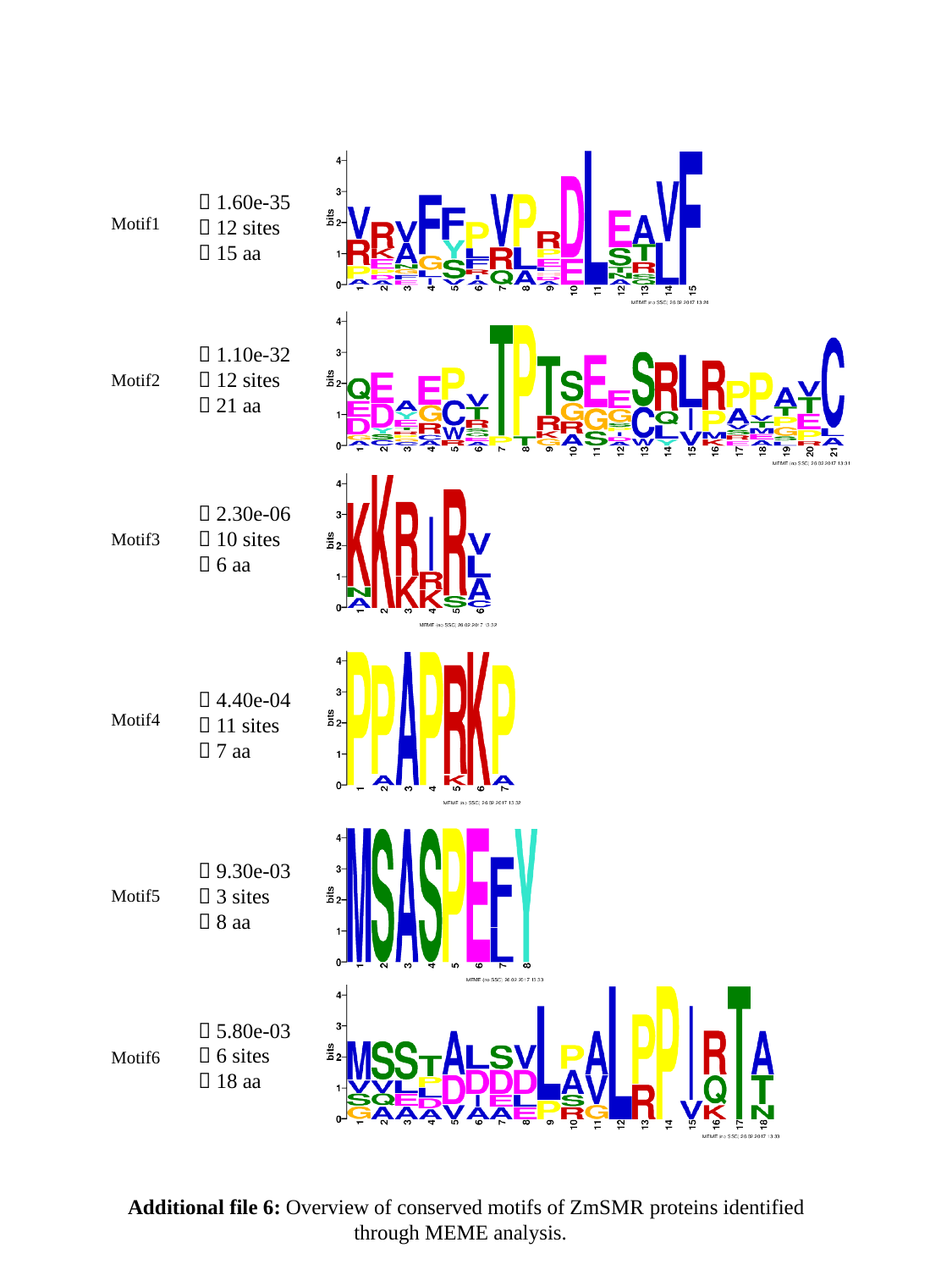

 1.60e-35
 12 sites
 15 aa
 1.10e-32
 12 sites
 21 aa
 2.30e-06
 10 sites
 6 aa
 4.40e-04
 11 sites
 7 aa
 9.30e-03
 3 sites
 8 aa
 5.80e-03
 6 sites
 18 aa
Motif1
Motif2
Motif3
Motif4
Motif5
Motif6
Additional file 6: Overview of conserved motifs of ZmSMR proteins identified
through MEME analysis.
