# Supplementary material for: Genome-wide evolutionary characterization and expression analysis of SIAMESE-RELATED family genes in maize
Source: BMC Evol Biol. 2020 Jul 29;20:91. doi: 10.1186/s12862-020-01619-2 (PMC7389639; doi:10.1186/s12862-020-01619-2)
Supplement: Supplementary file 8 — Additional file 8 Number of SMR in maize, Brachypodium distachyon, barley, millet, sorghum, soybean, Populus trichocarpa, rice, Arabidopsis. [file 12862_2020_1619_MOESM8_ESM.docx]

Additional file 8: Number of SMR in *Zea mays, Arabidopsis thaliana*, *Oryza sativa*, *Brachypodium distachyon*, *Setaria italica*, *Sorghum bicolor*, *Glycine max*, *Populus trichocarpa* and *Physcomitrella patens.*

| Name | Group I | Group II | Group III | Group IV | Group V | Group VI | Group VII | Total |
| --- | --- | --- | --- | --- | --- | --- | --- | --- |
| ZmSMR | 6 | 2 | 3 | 1 | 0 | 0 | 0 | 12 |
| BdSMR | 7 | 3 | 3 | 2 | 0 | 1 | 0 | 17 |
| SiSMR | 1 | 0 | 1 | 3 | 1 | 1 | 2 | 9 |
| SbSMR | 4 | 2 | 2 | 1 | 1 | 0 | 0 | 10 |
| GmSMR | 3 | 1 | 0 | 0 | 3 | 1 | 3 | 11 |
| PtSMR | 3 | 0 | 4 | 2 | 1 | 1 | 0 | 11 |
| OsSMR | 2 | 1 | 2 | 1 | 4 | 1 | 0 | 11 |
| AtSMR | 2 | 2 | 0 | 2 | 2 | 0 | 8 | 16 |
| PpSMR | 0 | 0 | 0 | 0 | 1 | 9 | 0 | 10 |
